# Prevalence, characteristics, and risk of exacerbation in young patients with chronic obstructive pulmonary disease

**DOI:** 10.1186/s12931-022-02144-0

**Published:** 2022-08-22

**Authors:** Yong Suk Jo, Kyung Joo Kim, Chin Kook Rhee, Kwang Ha Yoo, Ki-Suck Jung, Yong-Bum Park

**Affiliations:** 1 Division of Pulmonary and Critical Care Medicine, Department of Internal Medicine, College of Medicine, Seoul St. Mary’s Hospital, The Catholic University of Korea, Seoul, South Korea; 2 Division of Pulmonary and Allergy Medicine, Department of Internal Medicine, Konkuk University School of Medicine, Seoul, Republic of Korea; 3 Division of Pulmonary Medicine, Department of Internal Medicine, Hallym University Sacred Heart Hospital, Hallym University Medical School, Anyang, Korea; 4 Division of Pulmonary, Allergy, and Critical Care Medicine, Department of Internal Medicine, Hallym University Kangdong Sacred Heart Hospital, Seoul, Korea

**Keywords:** Young patients with COPD, Smoking, Exacerbation, Medical cost

## Abstract

**Background and objective:**

Early identification of chronic obstructive pulmonary disease (COPD) in young individuals could be beneficial to attempt preventive interventions. The objective of this study was to investigate clinical features and outcomes of young individuals with COPD from the general population cohort.

**Methods:**

We included individuals from the Korean National Health and Nutrition Examination Survey (KNHANES) with spirometry and identifiable smoking status. Young subjects with COPD were defined as aged between 40 and 50 years and had baseline forced expiratory volume in 1 s [FEV_1_]/forced vital capacity [FVC] ratio less than 0.7. Outcomes include the risk of exacerbation and medical expenses during 3 years of follow-up.

**Results:**

Among 2236 individuals aged between 40 and 50 years, 95 (4.2%) had COPD, including 36 who were never-smokers and 59 who were ever-smokers. Approximately 98% of COPD subjects had mild to moderate airflow limitation. Inhaler treatment was given to only 6.3% patients in the COPD group. The risk of exacerbation for a 3-year period was analyzed using the never-smoker, non-COPD group as a comparator. Hazards ratio for exacerbation was 1.60 (95% confidence interval [CI] 0.18–14.20) in the never-smoker COPD group and 1.94 (95% CI 0.31–12.07) in the ever-smoker COPD group of young subjects. COPD related medical costs were not significantly different between non-COPD and COPD groups of young individuals.

**Conclusions:**

The risk of exacerbation showed an increasing trend in COPD patients regardless of smoking status compared to non-COPD. More attention to early identification and provision of preventive measures are needed to reduce disease progression and improve outcome.

## Introduction

Chronic obstructive pulmonary disease (COPD) is a common progressive disease that is characterized by persistent respiratory symptoms and airflow limitation due to airway and/or alveolar abnormalities. It is usually caused by significant exposure to noxious particles or gases. Although COPD is not a fully reversible disease, it is regarded as a preventable and treatable disease. To date, COPD has been considered a disease of the elderly. However, growing evidence has shown that COPD can begin in early life and develop over many years. Thus, the concept of early COPD has gained interest [1–5]. Furthermore, taking account for the prevalence, morbidity, and mortality of COPD with a high burden on clinical and healthcare resources [6, 7], early detection and preventive interventions are needed to delay progression of COPD.

Half of COPD cases are developed from those with under-growth and maturation of lung during early adulthood. Another half of COPD cases are attributed to accelerated decline of lung function [8]. It has been recognized that COPD can start early in life. Thus, many efforts have been made to define those patients [3, 9]. Some studies have used the term of “mild” airflow limitation as a surrogate for “early COPD” [4, 5]. However, mild airflow limitation can occur at any age. This term does not mean early disease. It rather refers to the severity of the disease. Definitions for early COPD have not been standardized yet. Recently, Martinez et al. [10] have redefined “young individuals with COPD” as an age-dependent term for patients with COPD (FEV_1_/FVC < 0.7) at age of 20–50 years independent of the severity of airflow limitation. This simple definition is anticipated to be helpful for screening young patients with early stage of COPD.

Both pharmacologic and non-pharmacologic preventive interventions may lead to better outcomes for young patients with COPD [11–13] than for old aged clinically diagnosed COPD patients, resultantly slowing down the progression of disease and reducing healthcare expenditures. However, it is extremely difficult to enroll young patients with COPD because they rarely exhibit COPD related symptoms and visit hospitals. Thus, there is a lack of clinical information about such subjects. Moreover, approximately two-thirds of patients at risk of COPD in many individuals of primary clinics based studies are underdiagnosed and untreated appropriately [14–18]. However, we have the opportunity to evaluate this group of patients through our unique national insurance system. Thus, the objective of the present study was to determine the prevalence of young patients with COPD and assess their clinical features and outcomes with focused on COPD exacerbation risk and healthcare expenses.

## Methods

### Data source

For this study, cross-sectional data from the Korean National Health and Nutrition Examination Survey (KNHANES) were used. The KNHANES provides nationwide statistical data on the Korean population’s health and diet conducted annually. We included participants from January 2008 to December 2009 as index cases. We then merged the Korean National Health Insurance (NHI) database from 2007 to 2012 to investigate clinical information for a year prior to enrollment with follow-up period of 3 years. South Korea implements a compulsory health insurance system for people. The NHI database provides nationwide data with regard to illness and healthcare utilization patterns [19].

### Definition of COPD in young patients

**Fig. 1:**
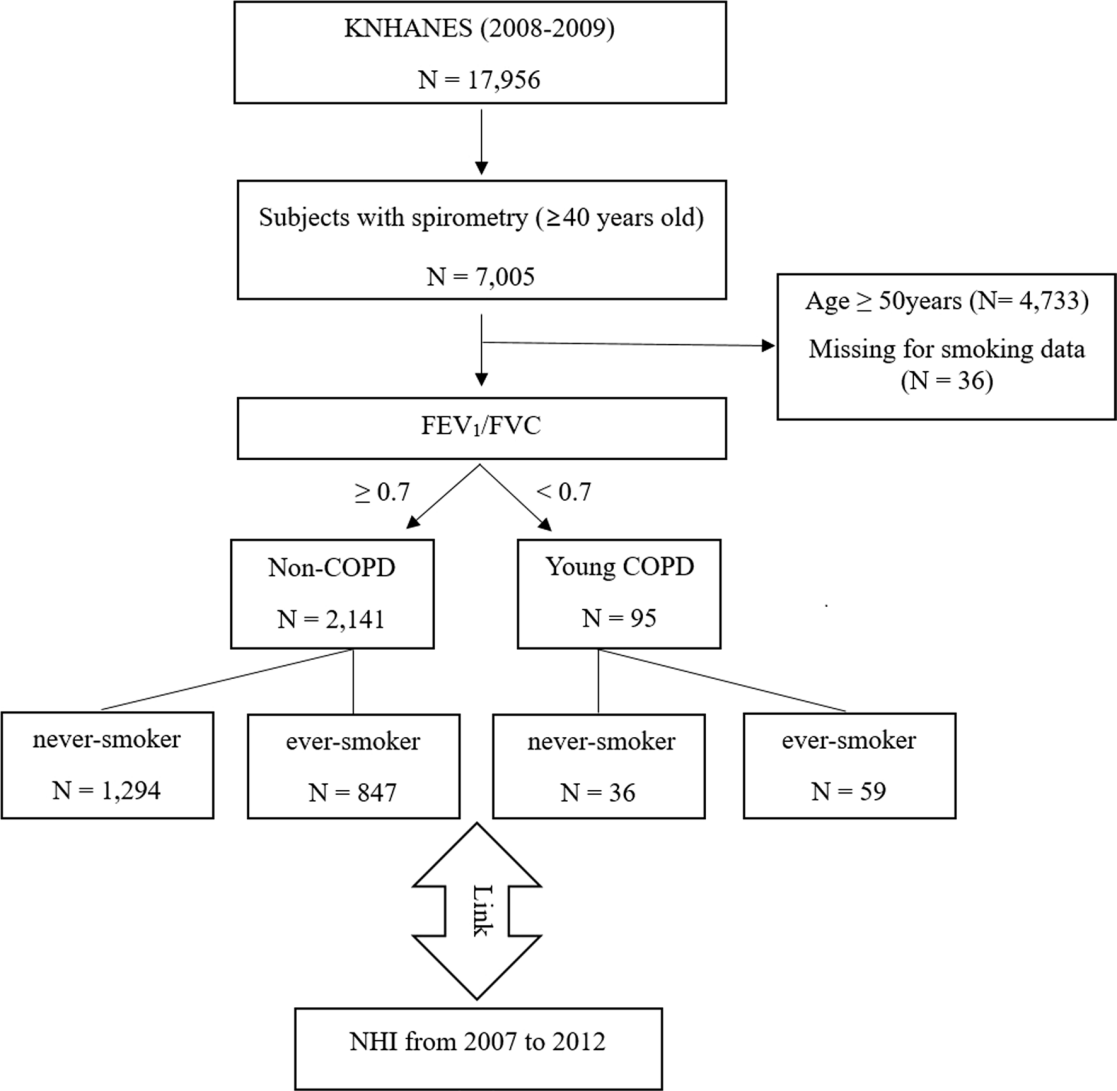
Study design. *COPD* chronic obstructive pulmonary disease, *FEV*_*1*_ forced expiratory volume in 1 s, *FVC* forced vital capacity, *KNHANES* Korean National Health and Nutrition Examination Survey, *NHI* National Health Insurance

Spirometry is only performed for individuals older than 40 years in KNHANES. Thus, we only included participants aged ≥ 40 years in our analyses. Subjects aged between 40 and 50 years whose spirometry showed airflow limitation (FEV_1_/FVC < 0.7) regardless of the severity of airflow limitation were defined as young patients with COPD [10]. Smoking status was classified into never-smoker and ever-smoker. In this study, we compared four groups classified by smoking status and COPD: (1) never-smoker, non-COPD, (2) ever-smoker, non-COPD, (3) never-smoker COPD in young patients, and (4) ever-smoker COPD in young patients (Fig. 1).

### Covariates

The KNHANES provides a variety of clinical information including demographic data (age, sex, body-mass index [BMI], education level, marital status, and self-perceived income status) and spirometry results. The Korean version of the EuroQoL-5 dimensions questionnaire (EQ-5D), a simple health-related quality of life instrument consisting of five health dimensions (mobility, self-care, usual activities, pain/discomfort, and anxiety/depression), was used to measure quality of life (QoL) status [20, 21]. Comorbid conditions were identified by searching for the International Classification of Disease, tenth Revision (ICD-10) codes in the Health Insurance Review and Assessment (HIRA) Service database.

### Outcome measurement

For this study, the primary outcome measure was moderate-to-severe exacerbation. A moderate exacerbation was defined as an outpatient clinic visit with an ICD-10 code for COPD (J43.x-J44.x, except J430) and a prescription of systemic steroids and/or antibiotics. Severe exacerbation was defined as an exacerbation necessitating hospitalization or an emergency department visit and prescription of systemic steroids and/or antibiotics. Another outcome measure was medical cost. We analyzed COPD associated medical costs extracted from the NHI database. All expenses are presented in US dollar (USD) with an exchange rate of 1 USD = 1211 Korean won (exchange rate on Jan 20, 2022).

### Statistical analysis

Clinical features among the four groups based on smoking and COPD status were compared using the Chi-square test for categorical variables and an analysis of variance for continuous variables. Exacerbation risk was compared by univariate and stepwise multivariate logistic regression analyses. Multivariable analyses were adjusted for covariates including age, sex, FEV_1_ of %predicted value, and prior exacerbation history. All analyses were two-sided and conducted at a significance level of 0.05 unless otherwise noted. All analyses were performed using SAS software, version 9.2 (SAS Institute Inc., Cary, NC, USA).

## Results

### Prevalence of young patients with COPD

Among 7005 adults with spirometry data, those aged over 50 years regardless of smoking status data were excluded. A total of 2236 subjects were then classified into non-COPD with FEV_1_/FVC ratio ≥ 0.70 (n = 2141, 95.8%) and young patients with COPD and FEV_1_/FVC ratio < 0.70 (n = 95, 4.2%). Both group of subjects were further classified depending on their smoking status into never-smoker and ever-smoker. Resultantly, four groups depending on COPD and smoking status were included in our final analyses: (1) never-smoker, non-COPD (n = 1294), (2) ever-smoker, non-COPD (n = 847), (3) never-smoker, COPD (n = 36), and (4) ever-smoker, COPD (n = 59) (Fig. 1).

### Clinical characteristics of young patients with COPD

Baseline characteristics are presented in Table 1. Comparison of baseline characteristics revealed that individuals with COPD had more past history of pulmonary tuberculosis and depression than individuals without COPD. Ever-smokers regardless of COPD had more males. Young individuals with COPD had lower FEV_1_%predicted value and lower FEV_1_/FVC ratio than young individuals without COPD. Although smoking pack-year was negatively correlated with FEV_1_ and FEV_1_/FVC ratio (correlation coefficient: − 0.12 and − 0.18, respectively; both *p* < 0.001), smoking status did not differ.

**Table 1:** Comparison of baseline characteristics of participants

	Non-COPD (n = 2141)		Young COPD (n = 95)		p value
	Never-smoker	Ever-smoker	Never-smoker	Ever-smoker	
Subjects (N)	1294	847	36	59	
Age, years	44.47 ± 5	44.37 ± 5	45.06 ± 6	44.98 ± 4	0.255
Sex, male (%)	163 (12.6)	761 (89.9)	7 (19.4)	55 (93.2)	< 0.001
BMI (kg/m^2^)	23.87 ± 3.98	24.65 ± 3.83	23.54 ± 4.58	23.74 ± 3.90	< 0.001
Smoking, pack-year	0	18.77 ± 15.65	0	22.52 ± 15.00	< 0.001
Comorbid condition					
DM	121 (9.4)	99 (11.7)	5 (13.9)	7 (11.9)	0.302
HTN	194 (15.0)	162 (19.1)	4 (11.1)	12 (20.3)	0.053
Ischemic heart disease	16 (1.24)	18 (2.13)	1 (2.78)	3 (5.1)	0.076
Congestive heart failure	9 (0.7)	4 (0.5)	0	1 (1.7)	0.630)
Gastroesophageal reflux disease	473 (36.6)	247 (29.2)	14 (38.9)	20 (33.9)	0.005
History of PTB	8 (0.6)	8 (0.94)	2 (5.6)	1 (1.7)	0.012
Depression	76 (5.9)	28 (3.3)	2 (5.6)	7 (11.9)	0.005
Atopic dermatitis	57 (4.4)	27 (3.2)	3 (8.3)	3 (5.1)	0.270
Any allergy^a^	521 (40.3)	295 (34.8)	15 (41.7)	18 (30.5)	0.045
Asthma	214 (16.5)	96 (11.3)	11 (30.6)	7 (11.9)	< 0.001
Index of quality of life, EQ-5D	0.97 ± 0.09	0.97 ± 0	0.95 ± 0.09	0.96 ± 0.09	0.224
Lung function					
FEV_1_, % predicted	93.79 ± 14.25	92.37 ± 13.42	72.91 ± 15.05	75.00 ± 14.81	< 0.001
FVC, % predicted	94.51 ± 14.32	93.38 ± 14.19	93.40 ± 15.42	94.27 ± 16.69	0.139
FEV_1_/FVC, %	0.83 ± 0.06	0.81 ± 0.07	0.65 ± 0.06	0.65 ± 0.05	< 0.001

Data are presented as number (%) or mean ± inter-quartile range

BMI, body mass index; DM, diabetes mellitus; GERD, gastroesophageal reflux disease; EQ-5D, EuroQol-5 dimensions questionnaire; FEV_1_, forced expiratory volume in 1 s; FVC, forced vital capacity; HTN, hypertension; ICS, inhaled corticosteroid; LABA, long-acting beta_2_ receptor agonist; LAMA, long-acting muscarinic receptor agonist; PTB, pulmonary tuberculosis; SABA, short-acting beta_2_ receptor agonist; SAMA, short-acting muscarinic receptor agonist

^a^History of allergic dermatitis/urticarial, contact dermatitis, food or drug allergy and allergic reaction were included

In the never-smoker group of COPD, 88.3% and 11.7% had Global initiative for COPD (GOLD 1, FEV_1_ ≥ 80%) and GOLD 2 (50 ≤ FEV_1_ ≤ 80%). None of the subjects had more than severe airflow limitation. On the other hand, 84.6%, 15.1%, and 0.3% in the ever-smoker group of COPD had GOLD 1, GOLD 2, and GOLD 3, respectively.

Prior history of asthma was found in 18 individuals: 11 of 36 (30.6%) in never smoker and 7 of 59 (11.9%) in ever-smoker group, but only 6 of those individuals were using inhaler treatment including ICS/LABA. Allergic traits also compared and atopic dermatitis and any allergy history were not different between non-COPD and young COPD group. On the other hand, a previous diagnosis of COPD was confirmed in only 6 of 95 young COPD group.

There were rare past exacerbation events (only 7 events in 2236 subjects) in both non-COPD and COPD groups. Inhaler treatment (inhaled corticosteroid and long-acting β2 agonist was prescribed in only 14 (0.63%) of 2,236 subjects and 6 (6.3%) of 95 with COPD).

### Risk of exacerbation

In young individuals with or without COPD, the risk of exacerbation during 3 years of follow-up is shown in Table 2. Exacerbation occurred in three young COPD subjects, and none of them received inhaler treatment. Incidence rate of exacerbation was 0.0069 per person-year in the non-COPD group and 0.0107 per person-year in the COPD group. Compared with the non-COPD group, the COPD group had higher risk of exacerbation after adjusting for age, sex, and FEV_1_, although the risk was not significantly higher (Hazard ratio (HR): 2.01; 95% confidence interval (CI) 0.49 to 8.17). For individuals classified by smoking and COPD status, HRs for exacerbation were 0.71 (95% CI 0.24–2.10), 1.60 (95% CI 0.18–14.20), and 1.94 (95% CI 0.31–12.07) for ever-smoker without COPD, never-smoker with COPD, and ever-smoker with COPD compared with never-smoker without COPD as a reference.

**Table 2:** Risk of moderate to severe exacerbation during a 3-year follow up

	Person-years	No of cases	Incidence rate (per person-year)	HR (95% CI)	
				Crude	Model^a^
COPD status					
None	6355.70	44	0.0069	Reference	Reference
Young COPD	280.67	3	0.0107	1.30 (0.40–4.29)	2.01 (0.49–8.17)
COPD and smoking status					
Never-smoker, non-COPD	3832.77	33	0.0086	Reference	Reference
Ever-smoker, non-COPD	2522.93	11	0.0044	1.10 (0.55–2.21)	0.71 (0.24–2.10)
Never-smoker, Young COPD	107.07	1	0.0093	0.93 (0.13–6.93)	1.60 (0.18–14.20)
Ever-smoker, Young COPD	173.61	2	0.0115	1.70 (0.40–7.27)	1.94 (0.31–12.07)

^a^Adjusted by age, sex and FEV_1_ (%)

### Disease related medical expenses

There was no significant difference in the cost of disease-related hospitalization or outpatient visits between non-COPD and COPD in young patients. Total cost per person-year was 1085.21 ± 954.61 USD vs. 1259.96 ± 1362.40 vs. 1005.52 ± 1189.53 USD for non-COPD vs. never-smoker COPD vs. ever-smoker COPD groups; *p* = 0.984). Detailed medical costs related to smoking and COPD are presented in Table 3. Compared to the non-COPD group, admission-related medical expenses were higher in the ever-smoker COPD group. However, overall medical expenses were similar between groups.

**Table 3:** COPD related medical expenses for 3 years follow up

	Non-COPD	Young COPD		P value
		Never-smoker	Ever-smoker	
COPD related medical expenses, cost per person-year				
Hospitalization	1779.92 ± 1289.52	1566.16 ± 1042.78	1849.75 ± 1001.13	0.997
Outpatient clinic	657.43 ± 610.12	848.42 ± 884.90	431.46 ± 531.47	0.357
Total	1085.21 ± 954.61	1259.96 ± 1362.40	1005.52 ± 1189.53	0.984

All costs are presented in US dollars (USD) with an exchange rate of 1 USD equal to 1211 Korean won (exchange rate on Jan 20, 2022)

Data are presented as cost per person-years ± standard deviation

## Discussion

Using a Korean population-based cohort with 2236 randomly selected individuals aged 40–50 years with spirometry and smoking data, the prevalence of young patients with COPD was 4.2% according to the definition by FEV_1_/FVC less than 0.7. Risk of exacerbation during a 3-year follow up in ever smokers without COPD and young patients with COPD regardless of smoking status tended to be higher compared to that for the non-smoker without COPD group. However, the risk was not significantly higher because the occurrence of an exacerbation event itself was very rare. Moreover, disease-related medical expenses were not significantly different according to smoking or COPD status.

The prevalence of COPD in young individuals aged between 40 and 50 years old in our study was 4.2% (95 of 2236 participants). In a nationally representative sample cohort of China, age-standardized prevalence of COPD in young individuals was 1.4% for age group of 20–29 years, 3% for age group of 30–39 years, 5.1% for age group of 40–49 years [22]. Both general population cohorts in China and Korea showed similar prevalence of spirometry-defined COPD in young participants.

Studies of mild or asymptomatic COPD with mild to moderate airflow limitation are rare, especially in young individuals because such patients generally do not have sufficient respiratory symptoms that would lead to a voluntary hospital visit. Therefore, it has been difficult to find and enroll these patients into trials or observational cohorts. However, we are able to assess those individuals through the KNHANES database which represents the general population in Korea. In our study, EQ-5D scores of COPD in young patients were almost normal. Most patients did not know their COPD status and were not given maintenance inhaler treatment from a clinician. They were found incidentally via spirometry screening. An extremely low prescription rate of inhaler therapy and normal ranged EQ-5D score indicated that these patients truly comprised of asymptomatic, mild COPD patients who had little motivation to visit clinics and follow up regularly.

Respiratory symptoms including chronic bronchitis (such as cough and phlegm) and shortness of breath are associated with increased risk of having airflow obstruction [23]. They are also associated with accelerated decline in lung function with − 2.71 ml/year excess decline in FEV_1_ and − 2.18 in FVC (*p* < 0.001 for both) as well as greater odds of incident airflow obstruction (odds ratio [OR]: 1.40; 95% confidence interval [CI] 1.24–2.14) [24]. This suggests that respiratory symptoms are among predictors for early identification of individuals who are at risk for developing COPD. Moreover, Woodruff et al. [25] showed that although subjects who do not meet diagnostic criteria for COPD, ever smokers with respiratory symptoms can experience exacerbations and activity limitation. Among 825 subjects of preserved pulmonary function, 18.9% had chronic bronchitis symptoms and 26.4% reported that they have diagnosed as COPD previously. As our study is not a multicenter observational cohort study, but general population based study, comprehensive, in-depth survey for respiratory symptoms might be limited. There is no available information on respiratory symptom of COPD in young patients in the KNHANES survey, but quality of life status was near normal and most subjects did not have history of previous COPD diagnosis in young COPD group. This suggestive of most young COPD subjects in this study might be asymptomatic and rarely visit hospital. Data of asymptomatic young COPD are rare, but we can approach those group of individuals through the KNHANES database.

It has become more evident that COPD can begin early in life and develop over many years [3, 26–29]. Individuals we encounter in the clinic are mainly older patients with a severe disease. Therefore, most researchers on COPD have focused on these patients. Identifying individuals who are likely to develop COPD at an early age could allow us to implement preventive interventions and resultantly delay progression, thereby reducing clinical and social burden. To date, the lack of a standardized definition for these group of COPD patients is regarded as one of main problems that hinder clinicians to focus on these patients. One international group of experts has proposed an operational definition of early COPD [2] and suggested that early COPD should be defined as individuals aged < 50 years with a smoking exposure more than 10 pack-years with one or more of the following: (1) FEV_1_/FVC less than the lower limit of normal (LLN), (2) abnormalities on chest CT compatible with COPD such as visual emphysema, air trapping, or bronchial wall thickening, and/or (3) accelerated FEV_1_ decline of more than 60 ml/year. Early COPD defined as FEV_1_/FVC less than the LLN in individuals under 50 years of age with more than 10 pack-years was reported in 15% of a general population cohort [30]. Another study defined early COPD with the same criteria except for smoking and found that 3% (168 of 5497 subjects) had early COPD and 12.5% of early COPD developed clinical COPD (FEV_1_/FVC of < 0.70 and FEV_1_ < 80% predicted) after 10 years compared to 1.6% of clinical COPD developed from non-early COPD [31]. Taking smoking into account, 24% of smokers with ≥ 10 pack-years, 10% of smoker with < 10 pack-years, and 3% of never smokers developed clinical COPD in those with early COPD.

We designed the present study based on recently proposed criteria for COPD in young patients [10] and found that the prevalence was 4.2%. However, when we applied LLN based criteria, 50.8% (1137 of 2236 individuals aged 40–50 years old) were classified as young patients with COPD. The possibility of over-estimation might be a problem when defining young patients with COPD by the LLN criteria. Fixed ratio (FEV_1_/FVC < 0.7) criteria has been used to identify airflow limitation but it would overdiagnose among older and underdiagnose among younger individuals [32, 33]. Colak et al., found that young and middle-aged adults with airflow limitation according to LLN (FEV_1_/FVC < LLN) but no fixed ratio criteria experienced increased morbidity and mortality [34]. This suggests the usefulness of patient surveillance through the LLN criteria to detect airflow limitation patients not confirmed by the fixed ratio criteria in young aged group. However, caution is required for overdiagnosis in young asymptomatic individuals as in our study population.

This study has merit as it is the first study to report the prevalence and clinical features of COPD in young patients of Korea based on the recently suggested diagnostic criteria. However, it also has some limitations. First, post-bronchodilator spirometry was not used to identify COPD in young patients at the baseline, nor we identified changes in lung function during the follow-up period. We defined COPD in young patients based on pre-bronchodilator spirometry that revealed FEV_1_/FVC < 0.70 irrespective of smoking status. Thus, some individuals might have a reversible airflow limitation indicating likelihood for asthma and might not have persistent airflow limitation on repeated examination. Indeed, to accurately define the COPD, post-bronchodilator test should be used, however it is difficult to perform post-bronchodilator spirometry test to perform on a general population of national samples. We assessed the risk of COPD related exacerbation and medical costs using a nationwide health service database based on ICD-10 codes for COPD and operational definition of exacerbation. Although we could not evaluate the progression of COPD in young patients into clinical COPD during three years of follow-up, exacerbation might reflect disease progression. In fact, the exacerbation risk increased in ever smoker young patients with COPD compared to non-smoker non-COPD patients. However, the risk was not statistically significant because the event itself was very rare. Second, although we showed exacerbations tended to increase in both never- and ever-smoker young COPD, caution is needed in interpretation because there were very few exacerbation events. Third, we assessed young patients with COPD aged 40–50 years because spirometry was allowed only for adults over the age of 40 in KNHANES. Therefore, there is a possibility of under-estimation for the prevalence of COPD in young patients. Third, we could not evaluate radiologic findings suggestive of COPD such as incidental emphysema or airway wall thickness on chest CT expressed as the square root of wall area of a 10-mm lumen perimeter (Pi10) and the 15th percentile method (Perc15) known to be associated with an increased risk for the development of airflow limitation [35].

In conclusion, we found 4.2% of individuals aged 40–50 years had COPD through the KNHNES survey which provides us a chance to evaluate asymptomatic, mild COPD in young individuals. Considering the high rate of under-diagnosed COPD in Korea [36], our results may also have the possibility of under-estimation. Although we did not find significant differences in exacerbation risk or healthcare cost between non-COPD and COPD groups of young individuals, there were trends of increased exacerbation risk in COPD young patients irrespective of smoking status. The prevalence was relatively low compared to that of over COPD, but considering clinical significance of this group of patients as disease progression, it should not be overlooked. Moreover, previous studies showed that younger patients could have better outcomes than older COPD patients [11, 12]. Thus, active surveillance for early identification of COPD in young individuals and initiate preventive strategies such as smoking cessation and bronchodilator treatment are needed to reduce disease progression and improve outcome.

## Data Availability

The data of KNHANES are publicly available at https://knhanes.kdca.go.kr/knhanes/eng/index.do, accessed on 15 Jan 2022.

## Abbreviations

COPD: Chronic obstructive pulmonary disease
FEV_1_: Forced expiratory volume in 1 s
FVC: Forced vital capacity
GOLD: Global Initiative for Chronic Obstructive Lung Disease
ICS: Inhaled corticosteroid
KNHANES: Korean National Health and Nutrition Examination Survey
LABA: Long-acting beta2 receptor agonist
LAMA: Long-acting muscarinic receptor agonist
mMRC: Modified Medical Research Council
6MWD: 6-Min walk distance
